# Constitutive expression of the global regulator AbrB restores the growth defect of a genome-reduced *Bacillus subtilis* strain and improves its metabolite production

**DOI:** 10.1093/dnares/dsac015

**Published:** 2022-05-24

**Authors:** Junya Yamamoto, Onuma Chumsakul, Yoshihiro Toya, Takuya Morimoto, Shenghao Liu, Kenta Masuda, Yasushi Kageyama, Takashi Hirasawa, Fumio Matsuda, Naotake Ogasawara, Hiroshi Shimizu, Ken-ichi Yoshida, Taku Oshima, Shu Ishikawa

**Affiliations:** 1 Graduate School of Science, Technology and Innovation, Kobe University, Nada, Kobe 657-8501, Japan; 2 Graduate School of Biological Sciences, Nara Institute of Science and Technology, Ikoma, Nara 630-0192, Japan; 3 Department of Bioinformatic Engineering, Graduate School of Information Science and Technology, Osaka University, Suita, Osaka 565-0871, Japan; 4 Biological Science Laboratories, Kao Corporation, Akabane, Tochigi 321-3497, Japan; 5 School of Life Science and Technology, Tokyo Institute of Technology, Yokohama, Kanagawa 226-8501, Japan; 6 Department of Biotechnology, Toyama Prefectural University, Imizu, Toyama 939-0398, Japan

**Keywords:** silencing, AbrB, global regulator, genome reduction, *Bacillus subtilis*

## Abstract

Partial bacterial genome reduction by genome engineering can improve the productivity of various metabolites, possibly via deletion of non-essential genome regions involved in undesirable metabolic pathways competing with pathways for the desired end products. However, such reduction may cause growth defects. Genome reduction of *Bacillus subtilis* MGB874 increases the productivity of cellulases and proteases but reduces their growth rate. Here, we show that this growth defect could be restored by silencing redundant or less important genes affecting exponential growth by manipulating the global transcription factor AbrB. Comparative transcriptome analysis revealed that AbrB-regulated genes were upregulated and those involved in central metabolic pathway and synthetic pathways of amino acids and purine/pyrimidine nucleotides were downregulated in MGB874 compared with the wild-type strain, which we speculated were the cause of the growth defects. By constitutively expressing high levels of AbrB, AbrB regulon genes were repressed, while glycolytic flux increased, thereby restoring the growth rate to wild-type levels. This manipulation also enhanced the productivity of metabolites including γ-polyglutamic acid. This study provides the first evidence that undesired features induced by genome reduction can be relieved, at least partly, by manipulating a global transcription regulation system. A similar strategy could be applied to other genome engineering-based challenges aiming toward efficient material production in bacteria.

## 1. Introduction

Notable progress in systematic genome reduction by genome engineering (hereafter referred to as ‘genome reduction’) has been achieved in the model bacteria *Bacillus subtilis* and *Escherichia coli* for nearly two decades.^1^ Because deleting large non-essential parts of genomes may remove competing and unwanted metabolic pathways that divert cell resources from the production of desired end products, such genome-reduced strains may have increased capacity for the production of desired metabolites and reduced impact of cellular burden, along with greater robustness and increased energy efficiency for a wide range of applications.^2^ Indeed, several genome-reduced strains that have beneficial traits compared with their parental wild-type strains have been reported to date. For example, *B. subtilis* MGB874 showed improved production of secreted heterologous enzymes.^3^^,^^4^ The production and secretion of *Staphylococcus aureus* antigens and lantibiotics that are difficult to express in other *B. subtilis* strains was achieved in the MiniBacillus strain GP10.^5^^,^^6^*Escherichia coli* DGF-298 showed better growth fitness and cell yields compared with those of the wild-type strain.^7^

However, genome reduction can also result in undesirable phenotypes such as growth defects^8^ and aberrant cellular and nucleoid morphologies.^9^ In the case of *B. subtilis* GP10, despite acquiring a trait for effective production, genome reduction results in decreased growth rate compared with that of the parental strain, even when grown in a rich medium, likely resulting from deletion of a ribosomal RNA gene.^10^^,^^11^ In the genome reduction process of *E. coli* DGF-298, further deletion became difficult due to the problems in growth fitness and cell yield when the genome size approached 3 Mb, and construction of the DGF-298 strain required restoration of the *proVWX* hyperosmolarity regulator genes to maintain growth.^7^ Thus, given that large/extensive genome reduction is associated with tradeoffs of undesirable phenotypes caused by gene deletion and changes in gene expression, there is likely an optimal reduction size for creating beneficial strains, which can be used for research and technology development.

**Figure 1:**
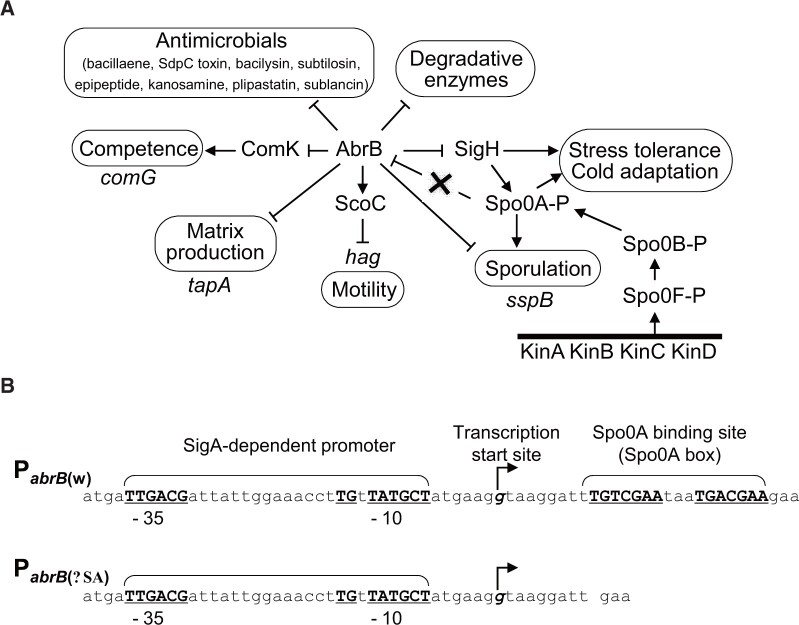
Transcriptional network centered on AbrB and the P_*abrB*__(ΔSA)_ mutation. (A) Spo0A is phosphorylated via a multicomponent system called phosphorelay from at least four sensor kinases (KinA, KinB, KinC, and KinD) to two phosphotranferases (Spo0F and Spo0B). Global gene silencing by AbrB is released by a decrease in the level of AbrB due to transcriptional repression of *abrB* caused by binding of Spo0A-P to the Spo0A box at the *abrB* promoter region. P_*abrB*__(ΔSA)_, an *abrB* promoter in which the Spo0A-binding sequence was deleted, is relieved from Spo0A-P-mediated repression without losing Spo0A function (indicated by a cross). Genes whose promoters used in this study are also indicated. (B) Sequences and features of the *abrB* promoter regions of the wild-type [P_*abrB*__(w)_] and the Spo0A box-deleted mutant [P_*abrB*__(ΔSA)_].

In the case of the *B. subtilis* MGB874 strain, deletion of 0.87 Mb (20%) of the genome reduced the growth rate, especially in minimal medium.^3^ In our preliminary transcriptome experiments, we found that AbrB-regulated genes were upregulated in strain MGB874 compared with those in the wild-type strain. AbrB is a global regulator that directly represses ∼250 genes involved in cell differentiation into several cell types with distinct phenotypes such as sporulating, genetically competent, motile, extracellular matrix-producing, and degradative enzyme-producing cells, which form multicellular communities known as biofilms on an agar plate or on a liquid surface (Fig. 1A).^12–14^ Cell differentiation is also observed in liquid culture in shaking flasks where the cells are kept dispersed.^15^ This process is initiated by a decrease in AbrB expression via transcriptional repression caused by binding of phosphorylated Spo0A to the promoter region immediately downstream of the transcriptional start site of *abrB* (Fig. 1B).^16^ Interestingly, in the *spo0A12* mutant strain, in which the 62nd codon of the *spo0A* gene is replaced by an amber mutation,^17^ AbrB is expressed at 2- to 4-fold higher levels than that in the wild-type strain, which was consistently found in various minimal media and accompanied by a significantly increased growth rate, whereas deletion of *abrB* decreased the growth rate compared with that of the wild-type strain.^18^ These findings suggest that, even in exponentially growing wild-type cells cultured in minimal medium, transcription of *abrB* is only partially repressed by Spo0A to induce a large number of AbrB-regulated genes, which would induce metabolic pathways that compete for cellular resources required in the pathway for the production of a desired end product (Fig. 1), consequently decreasing the growth rate. Therefore, further growth reduction would occur in the absence of AbrB, which fully induces the AbrB-regulated genes, and absence of the repressor Spo0A would increase the growth rate relative to that of the wild-type because high AbrB expression levels strictly repress AbrB-regulated genes. Considering these findings and presumptions, we hypothesized that constitutive expression of AbrB at a high level may rescue the reduced proliferation rate of MGB874.

Modulation of global transcription factors that could change global gene expression patterns offers a potential novel genome-wide approach to improve growth rate and material production in genome-engineered cells. In *E. coli*, overexpression of Lrp increases L-valine production^19^ and overexpression of modified H-NS improves acid tolerance.^20^ Lrp and H-NS are nucleoid-associated proteins,^21^ and our previous chromatin immunoprecipitation (ChIP)–chip and genome footprinting by high-throughput sequencing results suggest that AbrB binds to hundreds of sites on the *B. subtilis* genome and represses over 100 genes, similar to H-NS in *E. coli*.^13^^,^^14^ Exploration and visualization of single-molecule tracking data revealed the highly dynamic binding of AbrB throughout the genome, forming one or two regions of high-intensity binding on the nucleoids, similar to those of H-NS,^22^^,^^23^ which preferentially binds adenine and thymine-rich DNA to repress the expression of numerous genes.^24^ These data suggest that AbrB may silence a large number of genes simultaneously.

In this study, we investigated the effects of the constitutive high expression of *abrB* on the growth rate of the wild-type and MGB874 strains in minimal medium, along with its effects on the productivity of γ-polyglutamic acid (γ-PGA) and cellulase. This study provides the first demonstration that the problems induced by genome reduction can be solved, at least partly, by changing the expression level of a global transcription regulation system.

## 2. Materials and methods

### 2.1. Strain construction

The strains and plasmids used in this study are listed in Supplementary Table S1. The primers used for strain construction and verification are listed in Supplementary Table S2. The OA105 strain, in which two prophages (SPβ and PBSX), seven prophage-like regions (pro1 to pro7), and genes for the synthesis of plipastatin and bacillaene (*pps* and *pks*, respectively) were deleted, was used as the wild-type strain for most experiments instead of the parental 168 strain to avoid the interference of prophage induction and antibiotic production.^25^ Strains A-001 and A-024 were derived from strains 168 and MGB874 with restored tryptophan autotrophy, respectively. Construction of the strains and plasmids is detailed in the Supplementary Data.

### 2.2. Culture conditions

The strains were cultured in minimal medium buffered with MOPS (3-(*N*-morpholino)-propanesulfonic acid) (MMOPS medium, also referred to simply as minimal medium in this article) as previously described with the following modifications.^25^ First, glycerol stock was inoculated onto MYC solid medium [MMOPS medium supplemented with 10 g l^−1^ glucose, 1 g l^−1^ Bacto yeast extract (Difco Laboratories, Franklin Lakes, NJ, USA), and 0.4 g l^−1^ casamino acids (Difco Laboratories)] and cultured at 37°C for 10 h. For preculture, cells were collected from colonies, inoculated into 30 ml of MMOPS medium containing 10 g l^−1^ glucose to obtain an optical density at 600 nm (OD_600_) of 0.01, and cultured at 37°C with shaking at 200 rpm in a 300-ml flask until the OD_600_ reached 1–2. For the main culture, this preculture was inoculated in 50 ml of MMOPS medium with 10, 20, or 50 g l^−1^ glucose to obtain an OD_600_ below 0.1, followed by culturing at 37°C with shaking at 200 rpm in a 500-ml flask. Unless otherwise noted, 40 g l^−1^ CaCO_3_, which is soluble at acidic pH but hardly soluble at neutral pH, was added to maintain the culture medium at a neutral pH.

### 2.3. β-Galactosidase assay

To monitor β-galactosidase activity in liquid medium, the cells were grown in MMOPS medium containing 20 g l^−1^ glucose at 37°C with shaking, and 1 ml of culture was collected every 2 h to determine β-galactosidase activity, as previously described.^26^ To accurately measure galactosidase activity, CaCO_3_ was not added to the medium. Therefore, the pH decreased during the stationary phase, and the activity during the stationary phase was not measured.

### 2.4. Growth and fluorescence measurements for long-term culture

For long-term culture using a microplate reader (BioTek, Vinooski, VT, USA), MMOPS medium was used as described above but with the following modification. For the preculture, MYC solid medium containing 5 g l^−1^ glucose was used. Because CaCO_3_ could not be added to measure the OD_600_ and fluorescence in a microplate reader, MMOPS medium containing 20 g l^−1^ glucose was used to maintain a neutral pH under these conditions. For the main culture, the preculture was inoculated to obtain an OD_600_ of 0.02 in 0.15 ml of MMOPS medium, followed by culture in a 96-well black/clear bottom plate (Thermo Fisher Scientific, Waltham, MA, USA) at 37°C with double-orbital shaking at the ‘Fast’ speed setting, and OD_600_ (and fluorescence, if necessary) readings were taken every 0.5 h over a 45-h period. To ensure aeration and prevent the drying of culture media during long-term culture, the microplate was sealed with an oxygen-permeable transparent membrane (0.3-mm thick polydimethylsiloxane-non-coating sheet, VECELL Inc. Fukuoka, Japan).

### 2.5. Flow cytometric analysis

Flow cytometric analysis was carried out as described previously with the following modifications.^27^ Cells cultured in MMOPS medium in a microplate as described above were fixed with 4% (v/v) formaldehyde at 25°C for 30 min, washed once in a buffer (10 mM Tris-HCl [pH 8.0], 1 mM EDTA, 200 mM KCl, and 5% [v/v] glycerol) suspended in phosphate-buffered saline (1 mM KH_2_PO_4_, 3 mM Na_2_HPO_4_, and 155 mM NaCl), and analyzed by flow cytometry using CytoFlexS (Beckman Coulter, Brea, CA, USA) without sonication. Data analysis was performed using the CytoExpert software bundle. Green fluorescence was detected using a fluorescein isothiocyanate channel (525/40 BP) with an excitation beam at 488 nm. A total of 100,000 events were detected at a rate of <1,500 events s^−1^.

### 2.6. Transcriptome analysis

For transcriptome and metabolic analyses, MMOPS medium containing 10 g l^−1^ glucose was used, and 40 g l^−1^ CaCO_3_ was added to maintain the neutral pH of the culture medium. However, CaCO_3_ degrades RNA during the extraction process. Because we found that high-purity ultrafine CaCO_3_ particles (1–50 μm, Shiraishi Kogyo Kaisha, Ltd., Osaka, Japan) were resolved by mixing with EDTA, we used the ultrafine CaCO_3_ particles for RNA extraction, which were first resolved by mixing with an equal volume of 0.5 M EDTA (pH 8.0) for 3 min at room temperature. The cells were then collected by centrifugation at 5,000 ×*g* for 3 min, resuspended in a solution containing RNAprotect Bacteria Reagent (QIAGEN, Hilden, Germany), and 0.5 M EDTA at a 2:1 ratio, and incubated for 5 min at 25°C. Cells were pelleted at 15,000 ×*g* for 1 min and stored at –80 °C until use. To measure the OD_600_, cells and the CaCO_3_ suspension were mixed with five times the volume of HE buffer (0.1 M EDTA, 0.1 M HEPES (4-(2-hydroxyethyl)-1-piperazineethanesulfonic acid), and pH 7.0) to resolve the ultrafine CaCO_3_ particles in advance.

RNA extraction, synthesis of complementary DNA, terminal labeling, and hybridization with the oligonucleotide tiling chip were all performed according to the Affymetrix instruction manual, as previously reported.^28^ For transcriptome analysis, custom-designed Affymetrix GeneChips were used. Hybridization signal data, background correction, data normalization, and determination of expression levels of individual genes were all performed with the In Silico Molecular Cloning program, array edition (In Silico Biology), as previously described^29^ with small modifications. In this study, relative signal intensities were calculated by subtracting the signal intensities of mismatched probes from those of perfectly matched probes and then adjusted to confer a signal average of 500 in each experiment. Raw data (CEL format) from the present transcriptome have been deposited in the ArrayExpress database under accession number E-MTAB-11245. Functional categories of genes involved in the central metabolism pathway (CMP) were assigned according to the *Subti*Wiki database.^30^ The AbrB regulon includes seven transcriptional units that are known to be activated by AbrB, although the molecular mechanism is not clear; thus, only genes that are directly repressed by AbrB as listed in the *Subti*Wiki database (i.e. AbrB-regulated genes) were analyzed in this study. Differences in overall transcriptional changes in the CMP and AbrB-regulated genes, along with that in the genes involved in the biosynthesis of amino acids and purine/pyrimidine nucleotides were evaluated using a one-sided Mann–Whitney U-test with *P-*values < 0.05 considered statistically significant (Supplementary Table S3). Genes classified into both CMP-related genes and AbrB-related genes were excluded from this analysis. The *P*-values are shown in box and whisker plots. To extract differentially expressed (DE) genes between MGB874 and OA191 and to conduct functional enrichment analysis, the Kyoto Encyclopedia of Genes and Genomes pathway *for B. subtilis* 168^31^ and NetworkAnalyst tool were used.^32^ Our normalized data were log_2_-transformed, processed by the *limma* program,^33^ and visualized as a volcano plot; genes with log_2_-fold change ≥ 1 (upregulated) or ≤ 1 (downregulated) and *P *<* *0.05 were considered to be DE genes (Supplementary Table S3). Differentially up- and downregulated genes were also evaluated at a cutoff value of 1.5-fold change which detected a similar number of DE genes detected by the volcano plot analysis (Supplementary Table S3).

### 2.7. Measurement of extracellular glucose and metabolites

The glucose concentration in the culture supernatants was measured using an enzyme electrode glucose sensor (BF-5; Oji Scientific Instruments, Hyogo, Japan). Acetate, acetoin, and succinate concentrations in the culture supernatants were measured using a high-performance liquid chromatography system (Shimadzu, Kyoto, Japan) equipped with an ultraviolet–visible detector (210 nm) with TSKgel OApak-A and TSKgel OApak-P columns (Tosoh, Tokyo, Japan). The detailed method is described elsewhere.^34^ The specific rates of glucose consumption and acetate formation are defined as follows:

*d*[glucose]/*dt* = –ν*X*

*d*[acetate]/*dt* = ρ*X*

where *X* represents the biomass concentration [g-dry cell weight (DCW) l^−1^], and ν and ρ (mmol g-DCW^−1^ h^−1^) are calculated as the slopes of the regression lines between the integrated biomass concentration (g-DCW l^−1^ h) and glucose or acetate concentration (mmol l^−1^).^35^

### 2.8. Measurement of mass isotopomer distributions of proteinogenic amino acids

During the exponential phase (7.5–8 h), an appropriate amount of culture [OD_600_ × culture volume (ml) = 8] was collected via centrifugation. The cells were washed with 2 ml of 0.2 mol l^−1^ HCl to remove the CaCO_3_ and then further washed with 2 ml saline. The cells were hydrolyzed with 2 ml of 6 mol l^−1^ HCl at 105°C for 18 h. The samples were filtered through a Cosmonice filter W (0.45 µm; Nacalai Tesque, Kyoto, Japan), mixed with 10 µl of 600 µM cycloleucine, dried, and dissolved in 50 µl acetonitrile. After adding 50 µl of N-(*tert-tert-*butyldimethylsilyl)-N-methyl-trifluoroacetamide containing 1% *tert*-butyldimethylchlorosilane, the samples were incubated at 105°C for 1 h for derivatization. mass isotopomer distributions (MIDs) of proteinogenic amino acids were measured using a gas chromatograph/mass spectrometer (Agilent 7890A GC and 5975C Mass Selective Detector; Agilent Technologies, Santa Clara, USA) with a DB-5MS+DG column (Agilent Technologies). The detailed method is described elsewhere.^34^

### 2.9. ^13^C-Metabolic flux analysis

The CMP of *B. subtilis* was considered for flux estimation. An elementary metabolite unit framework was used to model the carbon atom transitions.^36^ All reactions, carbon atom transitions in CMP, and carbon atom transitions from precursor metabolites to amino acids are listed in Supplementary Table S4. Effluxes were determined from the measured specific rates and precursor requirements for the biomass synthesis of *B. subtilis*.^37^ The flux distribution was estimated to minimize the residual sum of squares between the measured and simulated MIDs of amino acids. Comparisons between measured and simulated MIDs are summarized in Supplementary Table S5. Goodness of fit was assessed using the chi-square test. The 95% confidence interval (CI) of each flux was calculated using a grid search algorithm.^38^ Calculations were performed using OpenMebius software^39^ in MATLAB R2014a (Mathworks, Natick, MA, USA).

### 2.10. Analyses of γ-PGA and cellulase production

Strains A-278 [A-024: *tufA*-*pgdS* (*spc*), *amyE*::P_*rrnO*_-*pgsBCAE* (*cat*), *ΔpgsBCAE-pgdS*] and A-306 [A-278: P_*abrB*__(__ΔSA__)_ (*erm*)] were grown in MMOPS medium supplemented with 50 g l^−1^ glucose and γ-PGA after 24 h of culture and analyzed as previously described.^40^ For cellulase production, the recombinant plasmid pHYS237 encoding a cellulase gene from *Bacillus* sp. KSM-S237^3^ was introduced into strains A-001 (168: *trp+*) and A-395 [A-001: P_*abrB*__(__ΔSA__)_]. The transformants were cultured in 2xL-Mal medium (2% tryptone peptone, 1% yeast extract, 1% NaCl, 7.5% maltose hydrate, and 7.5 μg ml^−1^ MnSO_4_) supplemented with 15 mg l^−1^ tetracycline for 72 h at 37°C, and cellulase activity in the culture medium was determined as previously described.^41^ Differences were assessed with one-sided Student’s *t*-tests, and *P*-values < 0.05 were considered statistically significant.

## 3. Results

### 3.1. Deletion of the Spo0A binding site at the *abrB* promoter rescues the reduced growth of the genome-reduced strain MGB874

**Figure 2:**
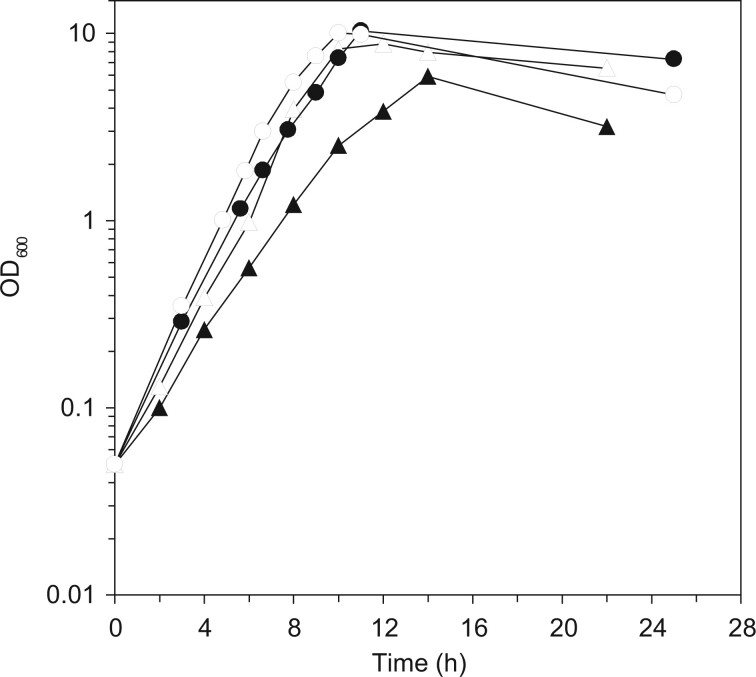
Growth of the wild-type and genome-reduced strain and their P_*abrB*__(ΔSA)_-introduced derivatives. Growth curves of the wild-type strain (OA105, closed circles) and its P_*abrB*__(ΔSA)_ derivative (OA190, open circles), the genome-reduced strain (MGB874, closed triangles) and its P_*abrB*__(ΔSA)_ derivative (OA191, open triangles) grown in the minimal medium containing 10 g l^−1^ glucose are shown.

To create cells that have a potential for effective production of useful products such as secreted proteins and γ-PGA, it is essential to identify and counter the cause of the slow proliferation of MGB874 cells. Our preliminary transcriptome experiments revealed that the expression of AbrB-regulated genes was upregulated compared with that in the wild-type when grown in the minimal medium containing glucose and ammonium as the sole carbon and the nitrogen sources, respectively. We assumed that the gene repression by AbrB was partially deregulated in MGB874 during the process of genome reduction and the upregulated AbrB-regulated genes (Fig. 1A) reduced the growth rate. Thus, we expected that constitutive expression of AbrB at high levels, as in the *spo0A*-null mutant,^18^ may rescue the slow-growth phenotype. Contrary to this expectation, *spo0A* deletion did not restore the slow-growth phenotype but rather showed an additional negative phenotype of a long lag time from inoculation to exponential growth in the minimal medium containing 20 g l^−1^ glucose (Supplementary Fig. S1). A deletion mutant of *sigH*, whose product activates *spo0A* transcription (Fig. 1A), also showed a similar long-lag phenotype. These results indicated that *spo0A* and *sigH* play important roles in the growth of MGB874 in minimal medium. SigH is required for the survival of *B. subtilis* in alkaline or acidic medium and for growth in ethanol-containing medium,^42^ and induction of Spo0A during exponential growth at 20°C is essential for stationary-phase viability in minimal medium (Fig. 1A).^43^ Thus, SigH and Spo0A are necessary for adaptation to various stresses and may also play an important role in growth in minimal medium with limited nutrient sources. Accordingly, to increase the growth rate of MGB874, we considered that the relief of AbrB expression from the Spo0A-mediadited repression without the loss of other Spo0A functions was required. To this end, we designed a promoter in which the Spo0A binding sequence, designated the Spo0A box, was deleted from the *abrB* promoter region [P_*abrB*__(ΔSA)_ mutation; Fig. 1B]. As expected, the growth rate of the MGB874 strain harboring the P_*abrB*__(ΔSA)_ mutation was restored to the level of the parental wild-type strain OA105 (Fig. 2).

### 3.2. P_*abrb*__(ΔSA)_ promoter is not repressed in minimal medium, thereby inhibiting the transcription of AbrB-regulated genes

**Figure 3:**
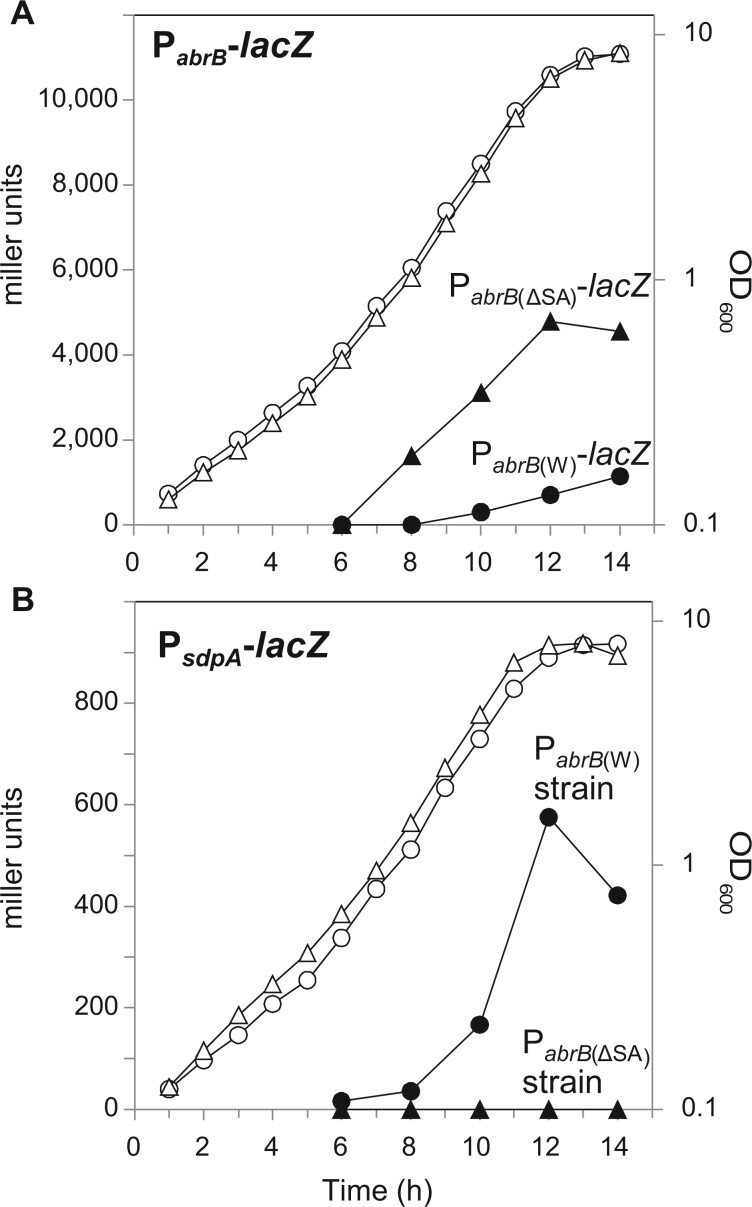
Effect of deletion of the Spo0A binding sequence at the *abrB* promoter. (A and B) LacZ assay of OA105-derived strains cultured in the minimal medium containing 20 g l^−1^ glucose. (A) The expression of P_*abrB*__(W)_-*lacZ* (OA124, closed circles) and P_*abrB*__(ΔSA)_-*lacZ* (OA125, closed triangles) and the growth of the OA124 (open circles) and OA125 (open triangles) strains carrying these promoter fusions are shown. (B) The expression of P_*sdpA*_-*lacZ* in P_*abrB*__(W)_ (OA131, closed circles) and P_*abrB*__(ΔSA)_ (OA137, closed triangles) and the growth of the OA131 (open circles) and OA137 (open triangles) strains carrying these promoter fusions are shown.

We predicted that, in minimal medium, transcription of *abrB* would be partially repressed by Spo0A to induce a large number of AbrB-regulated genes, imposing a burden for exponentially growing cells (see Introduction for details). To confirm this prediction and check whether P_*abrB*__(ΔSA)_ was relieved from Spo0A repression to supply enough AbrB to inhibit the expression of AbrB-regulated genes, we monitored *abrB* promoter activity using LacZ assays in the minimal medium containing 20 g l^−1^ glucose. The LacZ activity of the Spo0A box-deleted promoter fused with *lacZ* [P_*abrB*__(ΔSA)_-*lacZ*] and the wild-type promoter fused with *lacZ* [P_*abrB*__(W)_-*lacZ*] inserted in *the amyE* region on the chromosome was compared (Fig. 3A). P_*abrB*__(ΔSA)_-*lacZ* was active in the exponential phase, whereas the activity of P_*abrB*__(W)_-*lacZ* was repressed to a lower level. This result indicated that in minimal medium, Spo0A was activated to repress P_*abrB*__(W)_ in the exponential phase, whereas P_*abrB*__(ΔSA)_ was released from this repression.

To examine whether higher *abrB* transcription from P_*abrB*__(ΔSA)_ was sufficient to repress AbrB-regulated genes, the promoter activity of *sdpA*, which is repressed by direct AbrB binding and de-repressed in the absence of AbrB,^13^^,^^14^ was compared using P_*sdpA*_-*lacZ* in the P_*abrB*__(ΔSA)_ mutant and wild-type strains (Fig. 3B). In the wild-type strain, although P_*abrB*__(W)_-*lacZ* transcription was weakly induced during the exponential phase (at 10 h), P_*sdpA*_-*lacZ* transcription was induced during the exponential growth phase, indicating that P_*sdpA*_-*lacZ* was not repressed at this level of *abrB* expression. In contrast, in the P_*abrB*__(ΔSA)_ introduced strain [referred to as P_*abrB*__(ΔSA)_ strain], *abrB* showed a transcription level that was ∼6.7 times higher than that of the wild-type, as detected from the slopes of the P_*abrB*_-*lacZ* values (Fig. 3A), and P_*sdpA*_-*lacZ* continued to be almost completely repressed even in the transition phase (Fig. 3B). These results clearly indicated that, in the P_*abrB*__(ΔSA)_ strain, AbrB is highly expressed at the level sufficient to strongly repress AbrB-regulated genes.

### 3.3. P_*abrb*__(ΔSA)_ mutation represses genes involved in cell differentiation

**Figure 4:**
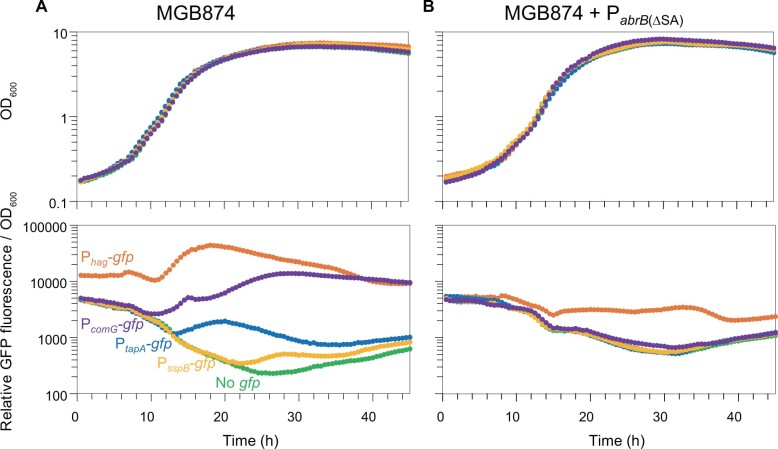
Effect of P_*abrB*__(ΔSA)_ on the activities of P*_hag_*-*gfp*, P*_tapA_*-*gfp*, P*_sspB_*-*gfp*, and P*_comG_*-*gfp*. (A) OA225 (MGB874 carrying P_*hag*_-*gfp*, orange), OA226 (MGB874 carrying P_*tapA*_-*gfp*, blue), OA227 (MGB874 carrying P_*sspB*_-*gfp*, yellow), OA228 (MGB874 carrying P_*comG*_-*gfp*, purple), and MGB874 (green) were grown in the minimal medium containing 20 g l^−1^ glucose, and the fluorescence intensity and OD_600_ were recorded every 30 min. (B) OA229 [MGB874 carrying P_*hag*_-*gfp*, P_*abrB*__(ΔSA)_, orange], OA230 [MGB874 carrying P_*tapA*_-*gfp*, P_*abrB*__(ΔSA)_, blue], OA231 [MGB874 carrying P_*sspB*_-*gfp*, P_*abrB*__(ΔSA)_, yellow], OA232 [MGB874 carrying P_*comG*_-*gfp*, P_*abrB*__(ΔSA)_, purple], and OA191 [MGB874 carrying P_*abrB*__(ΔSA)_, green] were grown and the fluorescence intensity and OD_600_ were recorded as in (A). (A color version of this figure appears in the online version of this article.)

During cultivation, small fractions of *B. subtilis* cells differentiate into the cells having different functions by expressing genes specific to each cell type.^12–14^ We monitored this process using a green fluorescent protein (GFP) reporter assay for differentiation-related genes. To observe whether the P_*abrB*__(ΔSA)_ mutation in the MGB874 strain represses genes involved in cell differentiation in minimal medium, the promoter activities of *hag*, *tapA*, *sspB*, and *comG*, which are specifically expressed only in motile cells, matrix-producing cells, sporulating cells, and competent cells, respectively (Fig. 1), were measured with a microplate reader using GFP as a reporter and monitored for 45 h. All examined promoters were activated in MGB874 but at different times (Fig. 4A). In contrast, in the P_*abrB*__(ΔSA)_ strain, while P_*hag*_-*gfp* exhibited weak activity, the activities of P_*tapA*_-*gfp*, P_*sspB*_-*gfp*, and P_*comG*_-*gfp* were almost completely repressed at all examined time points (Fig. 4B).

**Figure 5:**
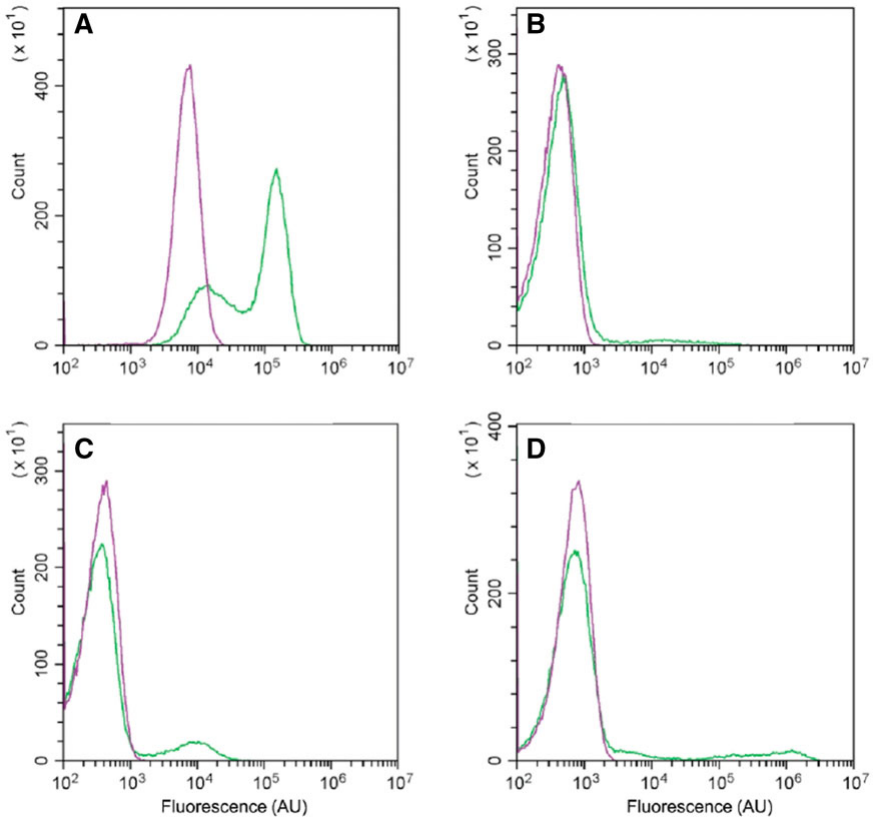
Flow cytometric analysis of the expression of P*_hag_*-*gfp* (A), P*_tapA_*-*gfp* (B), P*_sspB_*-*gfp* (C), and P*_comGA_*-*gfp* (D) in MGB874 (green line) and its P_*abrB*__(ΔSA)_-derivative (purple line). Cells were cultured as described in the legend to Fig. 4 for 20 h (A and B) or 30 h. (A color version of this figure appears in the online version of this article.)

Flow cytometric analysis using cells cultured under the same conditions was performed to examine the number of cells with such promoter activity in the population. Small portions of the population exhibited P_*tapA*_-*gfp*, P_*sspB*_-*gfp*, and P_*comG*_-*gfp* activities, whereas cells with activated P_*hag*_-*gfp* accounted for more than half of the population in MGB874 (Fig. 5). In contrast, in the P_*abrB*__(ΔSA)_-introduced MGB874 strain, the cells showed a single peak, indicating that P_*abrB*__(ΔSA)_ almost completely suppressed differentiation, leading to a homogeneous, undifferentiated cell population.

### 3.4. P_*abrb*__(ΔSA)_ mutation restores the decreased transcription of Central metabolic pathway genes in MGB874

**Figure 6:**
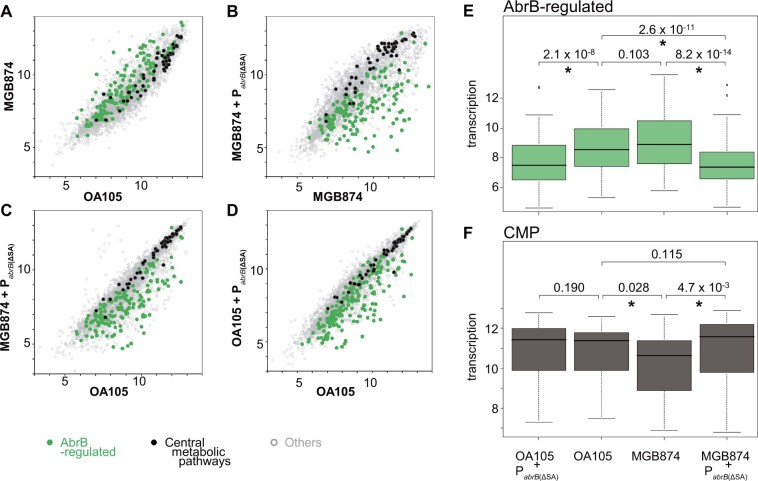
Comparison of the transcriptome profiles of AbrB-regulated and CMP genes between OA105 and MGB874 with or without the P_*abrB*__(ΔSA)_ mutation. (A–D) Scatter plots (log_2_ scale) of the transcriptional signals of each gene. AbrB-regulated (green) and CMP (black) genes are shown as colored dots. The other genes are shown as gray dots. (A) Scatter plots of OA105 and MGB874 cells. (B) Scatter plots of the MGB874 and MGB874 containing P_*abrB*__(ΔSA)_ strains (OA191). (C) Scatter plots of the OA105 and MGB874 containing P_*abrB*__(ΔSA)_ strains (OA191). (D) Scatter plots of the OA105 and OA105 containing P_*abrB*(ΔSA)_ strains (OA190). (E–F) Box plots (log_2_ scale) of transcription levels of AbrB-regulated and CMP genes in the OA105, MGB874, OA105 containing P_*abrB*__(ΔSA)_ (OA190), and MGB874 containing P_*abrB*__(ΔSA)_ (OA191) strains. Whiskers show minimum and maximum values, boxes represent the 25–75% data ranges, and horizontal lines within boxes are medians. *P* values calculated with one-sided Mann–Whitney U-test are shown. *P* values < 0.05 were considered to be significantly different (marked with an asterisk). (A color version of this figure appears in the online version of this article.)

We next examined how genome reduction and constitutive high expression of AbrB resulted in transcriptomic changes by performing a tilling array analysis. Figure 6 shows the transcription levels of all genes in exponentially growing cells in the minimal medium containing 10 g l^−1^ glucose for OA105, MGB874, and their corresponding P_*abrB*__(ΔSA)_-introduced strains.

The comparison of OA105 and MGB874 transcriptome profiles indicated that in the MGB874 strain, many AbrB-regulated genes were upregulated and many CMP-related genes were repressed (Fig. 6A). Although the overall transcriptional change of the AbrB-regulated genes was not statistically significant (Fig. 6E), 21% of the AbrB-regulated genes showed significant increase in expression level in MGB874 (Supplementary Table S3), as observed in preliminary transcriptome experiments. In contrast, the overall transcriptional reduction was statistically significant when comparing the repression of the CMP genes (Fig. 6F), and 21% of the CMP-related genes showed significant decrease in the expression level (Supplementary Table S3).

As described above, introduction of the P_*abrB*__(ΔSA)_ mutation into MGB874 restored the slow-growth phenotype to wild-type levels (Fig. 2A). This, in turn, resulted in a statistically significant difference in the overall transcription levels of AbrB-regulated and CMP genes (Fig 6E and F); 54% of the AbrB-regulated genes were statistically downregulated and 21% of the CMP genes were statistically upregulated compared with those of the parental strain MGB874 (Supplementary Table S3 and Figs 6B, E, and F). As a result, overall transcription level of CMP genes was restored to a level that was both visually and statistically indistinguishable from that of the wild-type strain OA105 (Fig. 6C and F).

**Figure 7:**
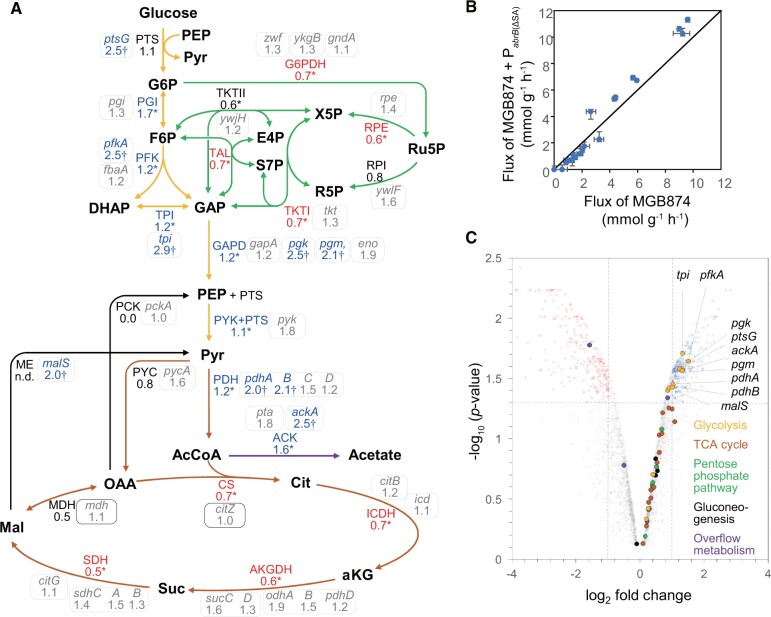
Metabolic flux distributions in the CMP in exponentially growing cells cultured in the minimal medium containing 10 g l^−1^ glucose in MGB874 and its P_*abrB*__(ΔSA)_-introduced derivative (OA191). (A) Relative values of the absolute flux values (mmol g^−1^ DCW h^−1^) of OA191 compared with those of MGB874 are shown beside the flux of the pathway indicated by the arrow. Asterisks indicate significant differences because the 95% CI does not overlap between the strains. Relative value of malic enzyme (ME) is shown as ‘n.d.’ because both flux values are zero. The enzymes catalyzing each reaction in the pathways were assigned according to the *Subti*Wiki database, and the fold change (without log_2_ transformation) in the transcription levels of the genes in OA191 compared with those in MGB874 are shown in enclosed boxes. DE genes, as judged by a volcano plot in (C), are marked with a dagger (†). (B) Absolute flux values (mmol g^−1^ DCW h^−1^) in the CMP of MGB874 compared with those of the P_*abrB*__(ΔSA)_ strain using scatter plots. (C) Volcano plot analysis of the gene expression levels between MGB874 and OA191. Dots for genes involved in glycolysis, PPP, TCA cycle, gluconeogenesis, and overflow metabolism in the CMP are separately colored as indicated in the inset. Genes with log_2_-fold change ≥ 1 (upregulated) or ≤ 1 (downregulated) and *P* < 0.05 were considered to be DE.

Gene set enrichment analysis of DE genes detected enrichment of various metabolic pathways (*P *=* *3.0 × 10^−4^). The dots for genes involved in glycolysis, pentose phosphate pathway (PPP), tricarboxylic acid (TCA) cycle, gluconeogenesis, and overflow metabolism in CMP are separately colored in the volcano plot between MGB874 and the P_*abrB*__(ΔSA)_-introduced MGB874 strain (OA191) (Fig. 7C). Although the increase in the expression level of only glycolysis genes was statistically significant, most CMP genes were upregulated in the P_*abrB*__(ΔSA)_-introduced strain (Figs 6B, 7A, and C).

### 3.5. P_*abrb*__(ΔSA)_ mutation increases glycolytic flux in MGB874

To investigate how metabolism changes along with changes in transcription, we also performed ^13^C-metabolic flux analysis simultaneously using the same culture medium that was used for the transcriptome analysis of the parent and P_*abrB*__(ΔSA)_-introduced MGB874 strains. After flux optimizations, the residual sums of squares between measured and simulated MIDs were 65.5 and 87.1 for the MGB874 and P_*abrB*__(ΔSA)_ strains, respectively. It suggests that the estimated flux distributions can explain the measured MIDs because the threshold of chi-squared test for goodness of fit was 118.8 (the number of independent data was 111, while the number of degrees of freedom of the reaction model was 16). The flux distributions of CMP are shown in Fig. 7A, and the associated 95% CIs are shown in Supplementary Table S6. The glycolytic flux was increased in the P_*abrB*__(ΔSA)_ strain. For example, in the glucose-6-phosphate isomerase reaction, the upstream step in glycolysis, the 95% CI was 3.8–4.5 mmol g-DCW^−1^ h^−1^ in the P_*abrB*__(ΔSA)_ strain and was 2.3–3.0 mmol g-DCW^−1^ h^−1^ in the MGB874 strain.

This change in glycolytic flux was consistent with that found for the transcription levels. Furthermore, the acetate overflow flux was increased in the P_*abrB*__(ΔSA)_ strain (Table 1), which is also consistent with the upregulation of *pta* and *ackA*, which are responsible for acetate overflow (Fig. 7A). In contrast, the fluxes of the PPP and TCA cycle of the P_*abrB*__(ΔSA)_ strain were lower than those of the MGB874 strain. The expression levels of the genes for these pathways were higher in the P_*abrB*__(ΔSA)_ stain than in the parent strain, but increases were statistically not significant. These differences explain the greater flux toward glycolysis and acetate overflow than the PPP and TCA cycle.

**Table 1:** Growth rate and glucose uptake, acetate production, and succinate production of MGB874 and its P_*abrB*__(ΔSA)_ strains

Strain	Growth rate (h^−1^)^a^	Specific rate (mmol g-DCW^−1^ h^−1^)^b^		
		Glucose uptake	Acetate production	Succinate production
MGB874	0.46 ± 0.01	5.9 ± 0.7	2.7 ± 0.6	0.2 ± 0.1
OA191 [MGB874: P_*abrB*__(ΔSA)_]	0.51 ± 0.04	6.7 ± 0.9	4.4 ± 0.2	0.2 ± 0.0
*P*-value	0.054	0.15	4.8 × 10^−3^	0.81

a The growth characteristics of the indicated strains in the minimal medium containing 10 g l^−1^ glucose over 5–6 h.

b The data are presented as the mean ± SD (*n* = 3).

### 3.6. P_*abrb*__(ΔSA)_ mutation increases the transcription of genes involved in the biosynthesis of purine/pyrimidine nucleotides and amino acids in MGB874

**Figure 8:**
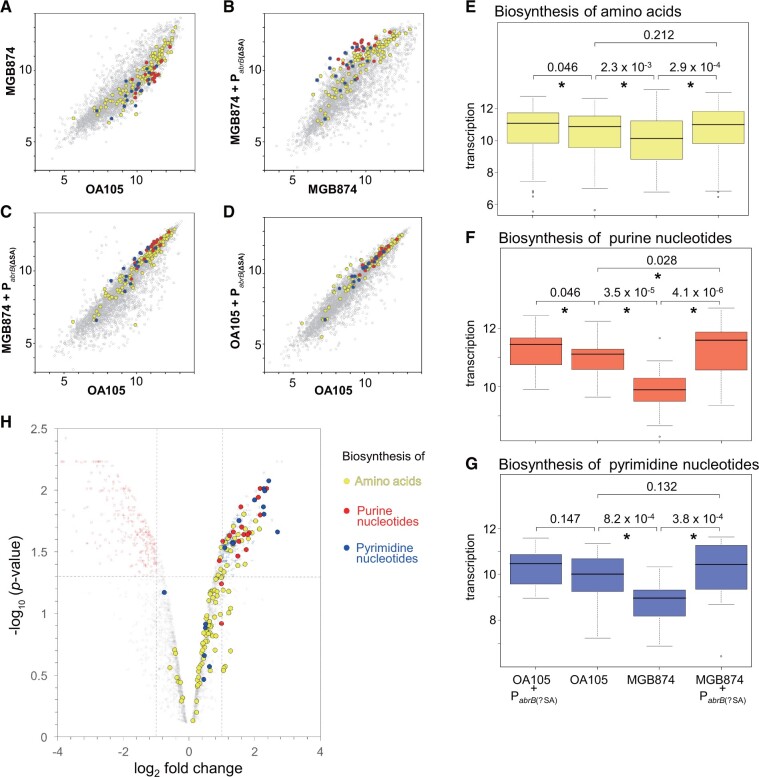
Comparison of transcriptome profiles of genes involved in the biosynthesis of amino acids and purine/pyrimidine nucleotides. (A–D) Scatter plots (log_2_ scale) of the transcriptional signals of each gene. Genes involved in the biosynthesis of amino acids and purine and pyrimidine nucleotides are indicated by yellow, red, and blue dots, respectively (CMP genes are included in ‘other genes’). (E–G) Box plots (log_2_ scale) of transcription levels of genes involved in the biosynthesis of amino acids and purine and pyrimidine nucleotides. Details are the same as described in the legend for Fig. 6E–F. (H) Volcano plot analysis of the gene expression levels between MGB874 and OA191. The genes involved in the biosynthesis of amino acids and purine and pyrimidine nucleotides are colored as in (A–D).

The functional enrichment analysis also detected enrichment of the pyrimidine and purine metabolism metabolisms (*P *=* *3.3 × 10^−6^ and 1.6 × 10^−5^, respectively) and of the alanine, aspartate, and glutamate metabolism (*P *=* *5.2 × 10^−5^). Thus, we further compared the transcriptional profiles of genes involved in biosynthesis of amino acids and purine/pyrimidine nucleotides using scatter plots (Fig. 8A–D), statistical evaluation of the overall transcriptional change (Fig. 8E–G), and volcano plots (Fig. 8H). The overall tendencies were similar to those found for the transcriptional changes of CMP genes. In all cases, the overall transcription levels were significantly lower in MGB874 than in OA105, and 80%, 83%, and 30% of the genes for biosynthesis of purines, pyrimidines, and amino acids, respectively, were significantly downregulated in MGB874 (Supplementary Table S3). Moreover, the introduction of P_*abrB*__(ΔSA)_ into MGB874 (strain OA191) restored the overall expression levels of these gene groups to the levels in OA105 (Fig. 8E–G) and significantly upregulated 85%, 67%, and 33% of the genes for biosynthesis of purines, pyrimidines, and amino acids, respectively (Fig. 8H and Supplementary Table S3).

### 3.7. P_*abrb*__(ΔSA)_ mutation enhances γ-PGA production

Although the MGB874 strain was unable to produce γ-PGA in MMOPS minimal medium, by placing the γ-PGA synthase genes (*pgsBCAE*) under the control of a strong promoter in the *amyE* region and deleting the genes from the original chromosome region, 2.2 g l^−1^ of γ-PGA was produced in the minimal medium containing 50 g l^−1^ glucose (Table 2). Additional introduction of the P_*abrB*__(ΔSA)_ mutation enhanced the production of γ-PGA to 4.1 g l^−1^, nearly doubling the yield. Considering that the OD_600_ of the P_*abrB*__(ΔSA)_ strain increased by 2-fold, these results indicated that the ability to produce γ-PGA has been almost doubled due to the restoration of growth rate by the introduction of P_*abrB*__(ΔSA)_. Thus, through genome reduction and constitutive high expression of AbrB, we were able to construct a strain with drastically enhanced productivity of desired substance.

**Table 2:** γ-PGA production in MGB874 and its P_*abrB*__(ΔSA)_ derivative

Strain	γ-PGA production^a^	
	U l^−1^	U l^−1^${\text{OD}}_{600}^{-1}$
A-278 (MGB874: γ-PGA production genes)^b^	2.2 ± 0.1 (100)	0.25 ± 0.014 (100)
A-306 [MGB874: γ-PGA production genes, P_*abrB*__(ΔSA)_]	4.1 ± 1.4 (189)	0.45 ± 0.032 (182)
*P*-value	0.042	9.8 × 10^−4^

a The data are presented as means ± SD (*n* = 3). Relative γ-PGA production compared with that of A-278 are indicated in parentheses.

b P_*rrnO*_-*pgsBCAE* (to overexpress the γ-PGA synthase genes) and *tufA-pgdS* (to overexpress a γ-glutamyl hydrolase gene to decrase culture viscosity and increase γ-PGA productivity) are inserted in the *amyE* region. The *pgsBCAE*-*pgdS* sequence in the original chromosome region has been deleted.

### 3.8. P_*abrb*__(ΔSA)_ mutation enhances cellulase productivity in the wild-type strain

We predicted that silencing the AbrB regulon by the P_*abrB*__(ΔSA)_ mutation would reduce the cellular burden, as in the case of genome reduction, leading to enhanced bioproduction. Although the introduction of this mutation did not change the growth rates of the wild-type strain OA105 or the 168 strain (Fig. 2 and data not shown), cellulase production in the minimal medium containing 50 g l^−1^ glucose, as determined based on the activities per culture volume or further normalized by OD_600_, increased by 1.7- and 2.0-fold, respectively, when the P_*abrB*__(ΔSA)_ mutation was introduced in the wild-type strain 168 (Table 3). This result indicated that the mutant strain has approximately double the ability to produce cellulase. Consistently, transcriptome analysis of OA105 and its P_*abrB*__(ΔSA)_-introduced derivative revealed statistically significant global transcriptional repression of AbrB-regulated genes in the P_*abrB*__(ΔSA)_-introduced OA190 strain (Fig. 6D and E), with 56% of the genes being downregulated (Supplementary Table S3). Although there was no apparent overall or statistically significant transcriptional change of genes for the CMP or biosynthesis of pyrimidine nucleotides (Figs 6D, F, 8D, and G), significantly increased transcription was detected for genes involved in the biosynthesis of amino acids and purine nucleotides (Fig. 8E and F), which may have contributed to increased metabolite productivity.

**Table 3:** Cellulase activities

Strain	Cellulase activities^a^			
	U l^−1^		U l^−1^${\text{OD}}_{600}^{-1}$	
168 (pHYS237)	2787 ± 136 (100)		107 ± 17 (100)	
168: P_*abrB*__(ΔSA)_ (pHYS237)^b^	4691 ± 84 (168)		217 ± 20 (203)	
*P*-value	3.3 × 10^−5^		1.0 × 10^−3^	

a The data are presented as the mean ± SD (*n* = 3). Relative cellulase activities compared with that of 168 (pHYS237) are indicated in parentheses.

b A-395 strain, a 168-derivative carrying the P_*abrB*__(ΔSA)_ mutation.

## 4. Discussion

The soil-dwelling bacterium *B. subtilis* has evolved sophisticated regulatory networks to differentiate cells into distinct subpopulations present in multicellular communities.^12^^,^^44^^,^^45^ During the exponential growth phase, the genes required for this cell differentiation are strictly repressed by AbrB.^12–14^ Under laboratory conditions, such genes are strictly repressed in a rich medium. However, in minimal medium, we found that the *sdpA* promoter was induced in the exponential growth phase (Fig. 3), indicating that the repression by AbrB was relieved, leading to induction of the AbrB-regulated genes, which may burden the cells. In the genome-reduced strain MGB874, AbrB-regulated genes were unexpectedly expressed more highly than those in the wild-type strain when grown in minimal medium.

Genome reduction is intended to remove redundant or less important genes with detrimental risk to reduce the aforementioned cellular burden. In fact, the MGB874 strain shows increased productivity of cellulase and protease^3^^,^^4^; however, this comes at a cost of a negative phenotype exhibiting slower growth on minimal medium. In this strain, 155 of the 249 genes regulated by AbrB remained, and this insufficient suppression by AbrB resulted in the significant upregulation of 21% of the 155 genes in the minimal medium compared with their expression in the wild-type strain OA105. Since the transcription level of *abrB* did not change in MGB874 (Supplementary Table S3), this partial derepression of AbrB-regulated genes could be caused by other mechanisms such as reduction of AbrB activity through AbrB phosphorylation by serine/threonine kinases^46^ or inhibition of AbrB activity through AbbA binding.^47^ Although it was not clear if one or both of these mechanisms is the primary cause, or if there are other reasons, we hypothesized that upregulation of some AbrB-regulated genes in MGB874 was the cause of the slower growth. In support of this hypothesis, introduction of the P_*abrB*__(ΔSA)_ mutation, which enables constitutive high expression of AbrB, restored the slow-growth phenotype in MGB874.

In the P_*abrB*__(ΔSA)_ strain, in addition to repression of the AbrB-regulated genes, the expression of genes involved in CMP and in the biosynthesis of amino acids and purine/pyrimidine nucleotides was restored to wild-type levels which was accompanied by an increase in the metabolic flux of glycolysis. These results suggested that in MGB874, the upregulation of AbrB-regulated genes may burden the cells and lead to the downregulation of genes for these metabolic pathways. Although it is unclear whether the effect of the upregulation of AbrB-repressed genes is direct or not, it is responsible for the slow-growth phenotype of MGB874 as this deleterious phenotype was ameliorated by introducing the P_*abrB*__(ΔSA)_ mutation.

In the P_*abrB*__(ΔSA)_ strain, P_*tapA*_-*gfp*, the promoter directly repressed by AbrB, along with the promoters indirectly repressed by AbrB, namely P_*hag*_-*gfp*, P_*sspB*_-*gfp*, and P_*comG*_-*gfp,* were repressed in minimal medium. This result indicates that the transcriptional network centered on AbrB was also tightly repressed in this strain, leading to the inhibition of cell differentiation to several cell types, which was observed in the parent MGB874. As such differentiation occurred in only a small fraction of cell population, it is unknown how much this inhibition contributed to the transcriptomic and metabolic changes that occurred in the P_*abrB*__(ΔSA)_-introduced strain. However, maintaining the cell population in an undifferentiated state may be important to improve its bioproductivity. In fact, the production of γ-PGA, which is produced from glucose via CMP in minimal medium containing glucose as the sole carbon source, was remarkably improved by introducing the P_*abrB*__(ΔSA)_ mutation (Table 2).

The bimodal expression pattern detected for P_*hag*_-*gfp* was previously reported in the undomesticated wild-type strain NCBI3610 using P*_hag_-yfp*.^48^ In this case, only the higher intensity peak was observed in its deletion mutant of *flgN* (*yvyG*), which encodes a chaperone protein essential for flagellar filament polymerization, and this shift was likely due to a change in regulation by the anti-sigma factor for SigD, FlgM. In contrast, we found that in the P_*abrB*__(ΔSA)_-introduced MGB874 strain, only the lower intensity peak was observed. In addition, the transcription levels of *flgN*, *flgM*, and *sigD* were not changed in the P_*abrB*__(ΔSA)_ strain, indicating that in the strain, P_*hag*_-*gfp* was repressed in a manner independent of these genes. It seems that ScoC is also not involved in the repression of P_*hag*_-*gfp*,^49^ because *scoC* expression was not upregulated in the P_*abrB*__(ΔSA)_ strain. Thus, it is unclear how constitutive overexpression of AbrB represses P_*hag*_, and further studies are needed to elucidate the mechanism.

Regarding cellulase production, it was reported that cellulase accumulated at a similar rate for up to 20 h to >2,000 U l^−1^ in both the 168 and MGB874 strains; however, while the production was arrested at this point in the 168 strain, it continued in the MGB874 strain increased by ∼2-fold.^3^ In this study, we showed that introduction of the P_*abrB*__(ΔSA)_ mutant into the wild-type 168 strain increased cellulase production by ∼2-fold at 72 h (Table 3), reaching a similar level to that of the MGB874 strain. This result supports the idea that tight repression of the transcriptional network centered on AbrB by the P_*abrB*__(ΔSA)_ mutation would reduce the cellular burden, similar to the effect of genome reduction in MGB874, leading to the enhancement of bioproduction, even in wild-type cells.

Based on previous findings for the wild-type strain JH642 with the *spo0A*-null mutation (*spo0A12*)^17^ (see Introduction for details), we initially expected that the increase in AbrB level would also increase the growth rates of the wild-type strains 168 and OA105 in minimal medium; however, the growth rate was not further increased. The downregulation of genes involved in CMP and the biosynthesis of amino acids and purine/pyrimidine nucleotides, which contributed to the decreased growth rate of MGB874, was not observed in the OA105 strain. Thus, the increase in AbrB level did not further increase the growth rate. Strains JH642 and 168 strains have 1,734 single-nucleotide polymorphisms (SNPs) in the 71-kb hypervariable region along with 35 SNPs distributed over the rest of the genome.^50^ These differences may be responsible for the reduced growth of JH642 in minimal media, and the increased AbrB level due to the *spo0A12* mutation may have restored the growth rate of JH642.

In summary, we have demonstrated that tight repression of a set of genes regulated by a global transcriptional regulator is an effective strategy for reducing the burden on cells to achieve higher bioproduction. This is probably effective in not only genome-reduced strains, in which a large number of unnecessary genes remain and/or the transcriptional network may be unexpectedly disturbed by genome engineering but also wild-type strains in which the transcriptional network may be disturbed under unnatural conditions for laboratory work or metabolite production. This is the first report of a method to improve the bioproductivity of genome-reduced strains by modifying a global transcription network, which can be developed as a key technology in the future not only *B. subtilis* and related species but also other bacteria with a mechanism to globally represses unwanted gene expression, such as the H-NS-mediated gene silencing in *E. coli*.^51^

## Supplementary data

Supplementary data are available at DNARES online.

## Supplementary Material

dsac015_Supplementary_Data
