# Supplementary material for: Constitutive expression of the global regulator AbrB restores the growth defect of a genome-reduced Bacillus subtilis strain and improves its metabolite production
Source: DNA Res. 2022 May 24;29(3):dsac015. doi: 10.1093/dnares/dsac015 (PMC9160880; doi:10.1093/dnares/dsac015)
Supplement: dsac015_Supplementary_Data [file dsac015_supplementary_data.zip › AbrB_Sup_text_20220516.docx]

**Supplementary data**

**Strain construction**

Strains, plasmids^1,2^, and primers used in this study are listed in Supplementary Tables S1 and S2, respectively.

**A-001 and A-024**

To restore tryptophan synthetic ability to the 168 and MGB874 strains, the *trpC* gene was amplified from *the Bacillus subtilis* ATCC6051 strain ^3^ with primer sets of trpC_F1/trpC_F2 and introduced into the 168 and MGB874 strains, and a non-auxotroph strain selected on minimal medium were designated as A-001 and A-024 strains.

**JA007**

To delete the *spo0A* gene, the upstream and downstream regions were amplified with primer sets spo0A-1s/spo0A-1r and spo0A-2f/spo0A-2s, respectively. The chloramphenicol-resistant cassette (*cat*) was amplified from pDLT3 ^4^ with the primer set rPCR-CmF2/rPCR-CmR. The three overlapping fragments were then ligated by overlap extension PCR (OE-PCR) with the primer set spo0A-1f/spo0A-2r and introduced into the A-024 strain.

**JA008**

To delete the *sigH* gene, the upstream and downstream regions were amplified with primer sets sigH-1s/sigH-1r and sigH-2f/sigH-2s, respectively. The chloramphenicol-resistant cassette (*cat*) was amplified as described above. The three overlapping fragments were then ligated by OE-PCR with the primer set sigH-1f/sigH-2r and introduced into the A-024 strain.

**OA020**

The Spo0A box in front of the *abrB* promoter was deleted as follows: three fragments were PCR-amplified from *B. subtilis* 168 using the following primer sets: metS-check-F/Dspo0Abox-FR, Dspo0Abox-PF/Dspo0Abox-PR, and Dspo0Abox-BF/yabC-check-R. The chloramphenicol-resistant cassette (*cat*) was amplified as described above. The four overlapping fragments were then ligated by OE-PCR with the primer set Dspo0Abox-FF/Dspo0Abox-BR and introduced into the 168 strain to create the 168-derived P*_abrB_*_(ΔSA)_ strain.

**OA021 and OA022**

The P*_abrB_*_(ΔSA)_ region of OA020 was amplified using the primer set abrBP-lacZ-F/abrBP-lacZ-R and placed upstream of *lacZ* of the OC14 strain (*amyE*::*lacZ* [*cat*]), which was created by the introduction of pLD2 ^2^, by a double-crossover event. The front and back fragments for recombination were amplified from the OC014 genome with primer sets lacZ-FF/lacZ-FR and lacZ-BF/lacZ-BR, respectively. The spectinomycin resistant cassette was amplified from pJL62 ^5^ with the primer set rPCR-specF/rPCR-specR. The four overlapping fragments were then ligated by OE-PCR and introduced into the OC014 strain to create the OA022 strain. As a control, the wild-type *abrB* promoter (P*_abrB_*_[W]_) was amplified from the 168 strain, and OA021 with P*_abrB_*_(W)_ was constructed in the same way.

**OA028**

The promoter region of *sdpA* was amplified from the 168 strain with the primer set sdpAP-lacZ-F/sdpAP-lacZ-R and integrated into the OC014 strain as described above.

**OA224**

The *gfpuv4* gene ^6^ was inserted into *the amyE* region with a spectinomycin cassette as follows. The *amyE* 5’ and 3’ regions for recombination for double crossover events were amplified with primer sets amyE-gfp-FF/amyE-gfp-FR and amyE-gfp-BF/amyE-gfp-BR, respectively. The chloramphenicol-resistant cassette (*cat*) was amplified as described above. The *gfpuv4* gene was amplified from pGFPuv4 using the primer set gfp-F/gfp-R. The four overlapping fragments were then ligated by OE-PCR with the primer set amyE-gfp-FF/amyE-gfp-BR and introduced into the A-024 strain.

**OA225**

To insert the promoter region of the *hag* gene, the *amyE* 5’ region and the *hag* promoter region were amplified from the 168 genome with primer sets amyE-gfp-FF/amyE-gfp-FR and Phag-F/Phag-R, respectively. The 5’ region of the *gfpuv4* gene was amplified from the OA224 genome with the primer set gfp-F-NoTag/gfp-BR1. The spectinomycin resistant cassette was amplified as described above. The four overlapping fragments were then ligated by OE-PCR with the primer set amyE-gfp-FF/gfp-BR1 and introduced into the OA224 strain.

**OA226**

To insert the promoter regions of *tapA*, the region was amplified from the 168 genome using the primer set PyqxM-F/PyqxM-R. The *amyE* 5’ region with the *spc* gene was amplified from the OA225 genome using the primer set amyE-gfp-FF/rPCR-specR. The 5’ region of the *gfpuv4* gene was amplified from the OA224 genome with the primer set gfp-F-NoTag/gfp-BR1. The three overlapping fragments were then ligated by OE-PCR with the primer set amyE-gfp-FF/gfp-BR1 and introduced into the OA224 strain.

To insert the promoter regions of *sspB* and *comGA*, the regions were amplified from the 168 genome with primer sets PsspB-F/PsspB-R and PcomGA-F/PcomGA-R, and OA227 and OA228 strains were similarly constructed as carried out for OA226.

**A-278**

Marker-free deletion was performed as previously described ^7^. For marker-free deletion of the *pgsBCAE* and *pgdS* regions, three fragments for recombination were amplified from the MGB874 genome with primer sets pgs-DFl/pgs-DRl, pgs-DF2/pgs-DR2, and pgs-lF/pgs-IR. A fragment for negative selection, the *mazF* cassette containing the spectinomycin resistance gene, was amplified from TMO310 with the primer set pAPNC-F/chpA-R. The three overlapping fragments were then ligated by OE-PCR with the primer set pgs-DFl/pgs-IR and introduced into the A-024 strain. After confirmation of no PCR error by sequencing, the marker region was removed as previously described.

The *pgdS* gene, which encodes the degradation enzyme for γ-PGA, was inserted downstream of the *tufA* gene as an operon to reduce the viscosity during γ-PGA production to increase productivity by enhancing aeration. Upstream and downstream regions from the 3’ end of *tufA* were amplified from the MGB874 genome using tufA-lf/tufA-lr and tufA-2f/tufA-2r, respectively. The spectinomycin resistant cassette was amplified as described above. The *pgdS* gene was amplified from the MGB874 genome using the primer set BSpgdS-F/BSpgdS-R. The four overlapping fragments were then ligated by OE-PCR with the primer set Psub-tufA-lf/tufA -2r and introduced into the *ΔpgsBCAE-pgdS* strain.

Finally, the γ-PGA synthetase gene *pgsBCAE*, under the control of the constitutive and strong *rrnO* promoter, was inserted in the *amyE* region. The 5’ region of *amyE* was amplified from the MGB874 genome using the primer set amyE-UF/amyE-UR. The *cat* cassette inserted in the *amyE* 3’ region was amplified from a pDLT3-introduced 168 genome ^4^ with the primer set cat-F/amyE-DR. The promoter region of *rrnO* was amplified from the RIK356 strain ^8^ using the primer set amyE-PrrnO-F/PrrnO-bs-pgsR. The *pgsBCAE* region was amplified from MGB874 using the primer set bs-pgsF/bs-pgs-catR. The four overlapping fragments were then ligated by OE-PCR with the primer set amyE-UF/amyE-DR and introduced into the strain carrying *ΔpgsBCAE-pgdS* and *tufA-pgdS*.

**References**

1. Morimoto, T., Kadoya, R., Endo, K., et al. 2008, Enhanced recombinant protein productivity by genome reduction in *Bacillus subtilis*. *DNA Res.*, **15**, 73-81.

2. Fukuchi, K., Kasahara, Y., Asai, K., Kobayashi, K., Moriya, S. and Ogasawara, N. 2000, The essential two-component regulatory system encoded by *yycF* and *yycG* modulates expression of the *ftsAZ* operon in *Bacillus subtilis*. *Microbiology*, **146 ( Pt 7)**, 1573-1583.

3. Albertini, A. M. and Galizzi, A. 1999, The sequence of the *trp* operon of *Bacillus subtilis* 168 (*trpC2*) revisited. *Microbiology*, **145**, 3319-3320.

4. Morimoto, T., Loh, P. C., Hirai, T., et al. 2002, Six GTP-binding proteins of the Era/Obg family are essential for cell growth in *Bacillus subtilis*. *Microbiology*, **148**, 3539-3552.

5. LeDeaux, J. R. and Grossman, A. D. 1995, Isolation and characterization of *kinC*, a gene that encodes a sensor kinase homologous to the sporulation sensor kinases KinA and KinB in *Bacillus subtilis*. *J. Bacteriol.*, **177**, 166-175.

6. Ito, Y., Suzuki, M. and Husimi, Y. 1999, A novel mutant of green fluorescent protein with enhanced sensitivity for microanalysis at 488 nm excitation. *Biochem. Biophys. Res. Commun.*, **264**, 556-560.

7. Morimoto, T., Ara, K., Ozaki, K. and Ogasawara, N. 2011, A simple method for introducing marker-gree deletions in the *Bacillus subtilis* genome. *Strain Engineering*, Springer, pp. 345-358.

8. Natori, Y., Tagami, K., Murakami, K., et al. 2009, Transcription activity of individual *rrn* operons in *Bacillus subtilis* mutants deficient in (p) ppGpp synthetase genes, *relA*, *yjbM*, and *ywaC*. *J. Bacteriol.*, **191**, 4555-4561.

**Figure Legends**

***Figure S1. Growth of genome reduction strain (MGB874) and MGB874-derived ΔsigH strain and Δspo0A strain.***

The cells were aerobically cultured in the minimal medium containing 20 g L^−1^ glucose in 96-well plate at 37˚C using microplate reader (Synergy H1, TioTek) to automatically measure OD_600_ for 48 hours.
