# Supplementary material for: Constitutive expression of the global regulator AbrB restores the growth defect of a genome-reduced Bacillus subtilis strain and improves its metabolite production
Source: DNA Res. 2022 May 24;29(3):dsac015. doi: 10.1093/dnares/dsac015 (PMC9160880; doi:10.1093/dnares/dsac015)
Supplement: dsac015_Supplementary_Data [file dsac015_supplementary_data.zip › AbrB_Sup_Figures_20220516.pptx]

## Slide 1
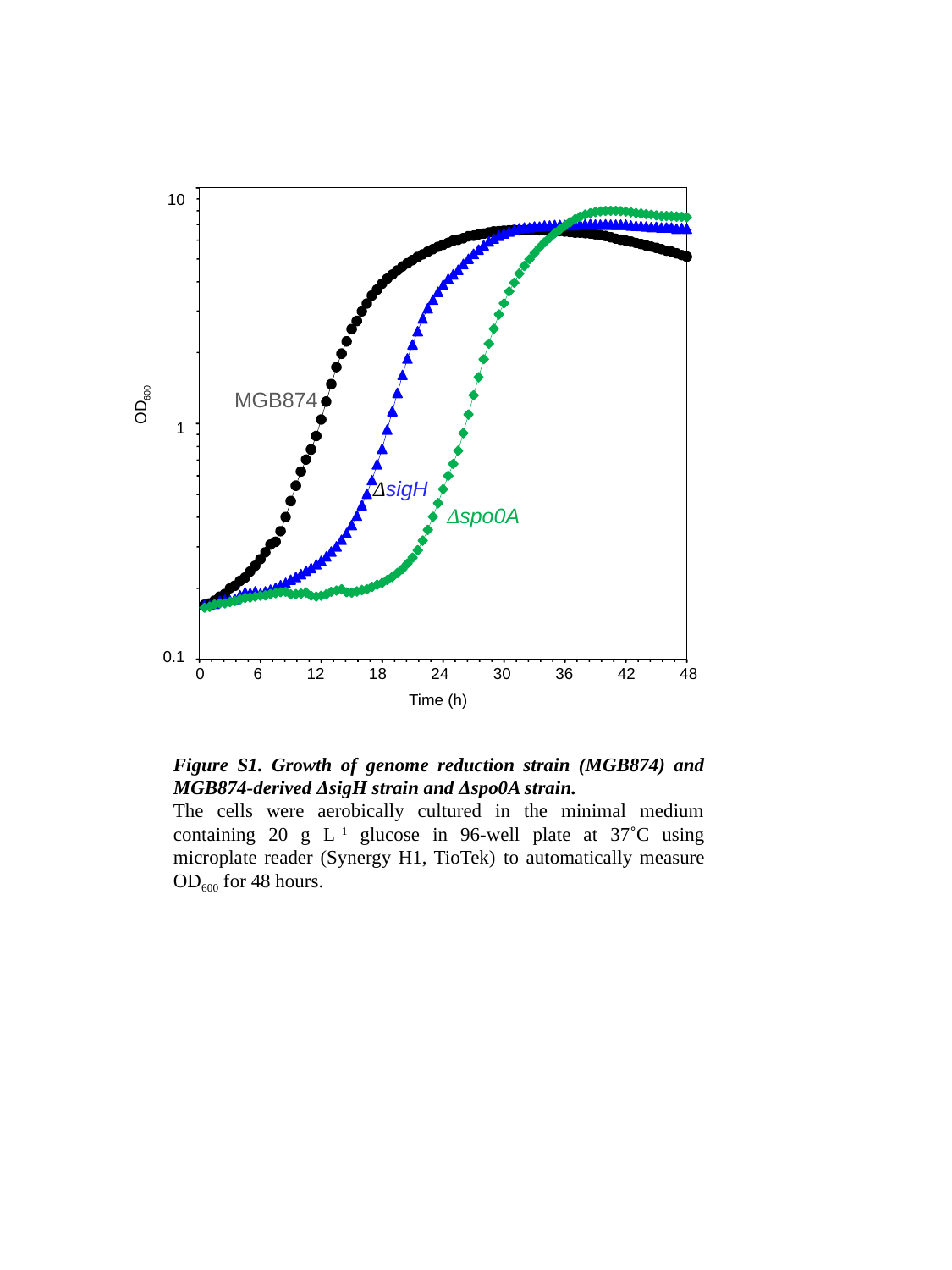

10
1
0.1
MGB874
ΔsigH
Δspo0A
OD600
0 6 12 18 24 30 36 42 48
Time (h)
Figure S1. Growth of genome reduction strain (MGB874) and MGB874-derived ΔsigH strain and Δspo0A strain.
The cells were aerobically cultured in the minimal medium containing 20 g L−1 glucose in 96-well plate at 37˚C using microplate reader (Synergy H1, TioTek) to automatically measure OD600 for 48 hours.
